# Pancreatic Neuroendocrine Tumors in Glucagon Receptor-Deficient Mice

**DOI:** 10.1371/journal.pone.0023397

**Published:** 2011-08-10

**Authors:** Run Yu, Deepti Dhall, Nicholas N. Nissen, Cuiqi Zhou, Song-Guang Ren

**Affiliations:** 1 Division of Endocrinology and Carcinoid and Neuroendocrine Tumor Center, Cedars-Sinai Medical Center, David Geffen School of Medicine at UCLA, Los Angeles, California, United States of America; 2 Department of Pathology, Cedars-Sinai Medical Center, Los Angeles, California, United States of America; 3 Department of Surgery, Cedars-Sinai Medical Center, and Department of Surgery, David Geffen School of Medicine at UCLA, Los Angeles, California, United States of America; 4 Division of Endocrinology, Diabetes, and Metabolism, Cedars-Sinai Medical Center, Los Angeles, California, United States of America; Ohio State University Medical Center, United States of America

## Abstract

Inhibition of glucagon signaling causes hyperglucagonemia and pancreatic α cell hyperplasia in mice. We have recently demonstrated that a patient with an inactivating glucagon receptor mutation (P86S) also exhibits hyperglucagonemia and pancreatic α cell hyperplasia but further develops pancreatic neuroendocrine tumors (PNETs). To test the hypothesis that defective glucagon signaling causes PNETs, we studied the pancreata of mice deficient in glucagon receptor (Gcgr^−/−^) from 2 to 12 months, using WT and heterozygous mice as controls. At 2–3 months, Gcgr^−/−^ mice exhibited normal islet morphology but the islets were mostly composed of α cells. At 5–7 months, dysplastic islets were evident in Gcgr^−/−^ mice but absent in WT or heterozygous controls. At 10–12 months, gross PNETs (≥1 mm) were detected in most Gcgr^−/−^ pancreata and micro-PNETs (<1 mm) were found in all (n = 14), whereas the islet morphology remained normal and no PNETs were found in any WT (n = 10) or heterozygous (n = 25) pancreata. Most PNETs in Gcgr^−/−^ mice were glucagonomas, but some were non-functioning. No tumors predominantly expressed insulin, pancreatic polypeptide, or somatostatin, although some harbored focal aggregates of tumor cells expressing one of those hormones. The PNETs in Gcgr^−/−^ mice were well differentiated and occasionally metastasized to the liver. Menin expression was aberrant in most dysplatic islets and PNETs. Vascular endothelial growth factor (VEGF) was overexpressed in PNET cells and its receptor Flk-1 was found in the abundant blood vessels or blood islands inside the tumors. We conclude that defective glucagon signaling causes PNETs in the Gcgr^−/−^ mice, which may be used as a model of human PNETs. Our results further suggest that completely inhibiting glucagon signaling may not be a safe approach to treat diabetes.

## Introduction

The pancreatic α cells are not well understood but have essential functions in normal physiology and diabetes pathogenesis [1], [2]. The main function of α cells is to secrete glucagon, an important hormone regulating glucose homeostasis. Glucagon signals through its receptor (GCGR), a G protein-coupled receptor, resulting in activation of the stimulatory G protein (Gs) and generation of cAMP [3]–[5]. The main target organ of glucagon is the liver where glucagon stimulates transcription and activity of key enzymes for glycogenolysis and gluconeogenesis, leading to increased hepatic glucose output. Abnormal glucagon signaling contributes to hyperglycemia of type 2 diabetes; glucagon signaling is thus an obvious target for treating type 2 diabetes [2], [6], [7]. Treatments designed to inhibit glucagon-GCGR interaction, such as GCGR antisense oligonucleotides, small-molecule GCGR antagonists, and GCGR-antagonizing antibodies, have demonstrated various benefits on diabetes in mice and primates [8]–[14]. Direct inhibition of glucagon signaling, however, raises safety concerns as it causes α cell hyperplasia, a possible precursor lesion of pancreatic neuroendocrine tumors (PNETs) [8]–[14].

Complete inhibition of glucagon signaling by genetic disruption of GCGR expression in mice (Gcgr^−/−^) lowers fasting and fed glucose levels but also results in hyperglucagonemia, α cell hyperplasia, and elevated levels of glucagon-like peptide 1 (GLP-1) [15], [16]. The hyperplastic α cells are kept in an immature phenotype, expressing markers of embryonic α cells such as GLUT2 [17]. The above phenotypes due to deficient GCGR are not limited to mice but are also present in humans. We have recently described for the first time a patient with a novel disease (Mahvash disease) of marked hyperglucagonemia without glucagonoma symptoms, diffuse α cell hyperplasia, and PNETs associated with a homozygous inactivating GCGR mutation (P86S) [18]–[20]. The 60-year-old patient was born to consanguineous parents. The P86S mutant exhibits abnormal receptor localization, lower glucagon binding capacity, and reduced cAMP production under physiological concentrations of glucagon. Furthermore, the P86S mutant fails to elicit calcium signal by glucagon and causes apoptosis. The patient's α cells also produced GLP-1 and to a lesser extent, pancreatic polypeptide. It is not clear if the patient's PNETs are caused by defective glucagon signaling due to the inactivating GCGR mutation. As the Gcgr^−/−^ mice exhibit improved glycemic control and recapitulate many of the phenotypes of animals treated with various methods of glucagon signaling inhibition and those of the patient with inactive, mutant glucagon receptor, they are a suitable model to test the hypothesis that defective glucagon signaling causes PNETs. We thus studied the pancreata of Gcgr^−/−^ mice and found 100% penetrance of PNETs in those mice at 10–12 months. Aberrant menin expression and angiogenesis likely mediate the PNET tumorigenesis. Thus complete inhibition of glucagon signaling indeed induces PNETs and its safety as a therapy for diabetes is questioned.

## Results

### Gross characteristics of the Gcgr^−/−^ mice

**Figure 1:**
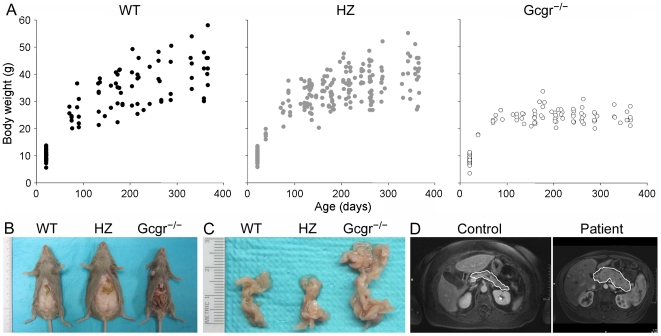
Gross characteristics of Gcgr^**−/−**^ mice. (A) Weight pattern from weaning to 12 months. Data were from 48 WT, 111 heterozygous, and 52 Gcgr^−/−^ mice. Body weight was measured periodically over time. (B) Abdominal visceral fat of 12-month WT, heterozygous, and Gcgr^−/−^ mice. (C) Representative pancreas of WT, heterozygous, and Gcgr^−/−^ mice at 12 months. Shown are pancreata after being fixed overnight in 10% formalin. (D) Pancreas appearance on MRI of an age- and sex-matched unrelated subject and the patient with homozygous inactivating P86S mutation of glucagon receptor. The pancreas contour was highlighted with a white line. HZ, heterozygous.

The Gcgr^−/−^ mice were born grossly normal and initially grew normally until at approximately 3 months, after when they did not gain significant weight as a group (Fig 1A). In contrast, the WT and heterozygous mice continued to gain weight throughout the observed 12 months and some became very obese. The Gcgr^−/−^ mice were lean, and the average weight of Gcgr^−/−^ mice at 10–12 months was 23.8±0.5 g, 40% lighter than those of WT (41.9±2.6) or heterozygous (39.8±1.4) mice (*p*<0.001) (Fig 1A and Table 1). Compared with their WT or heterozygous counterparts, the Gcgr^−/−^ mice did not exhibit obvious behavioral changes but tended to move less when closer to 12 months old. None of the 48 WT mice died during the observational period (12 months) while 4 of the 111 heterozygous mice (1 postpartum bleeding, 1 large pelvic tumor, and 2 unknown cause) and 3 of the 52 Gcgr^−/−^ mice (1 gross PNET and 2 unknown cause) died. The mortality rates of the 3 groups of mice were not significantly different. At both 2–3 months and 10–12 months, random glucose levels were significantly lower in Gcgr^−/−^ mice (154±9 and 96.2±9.0 mg/dl respectively) than those in WT (194±4 and 156.7±7.8) or heterozygous (191±19 and 156.2±5.5) mice (*p*<0.001) (Table 1). At 10–12 months, random glucagon levels remained markedly elevated (nearly 200-fold) in Gcgr^−/−^ mice compared with those in WT animals, while random insulin levels were slightly lower in Gcgr^−/−^ mice than in WT or heterozygous animals (Table 1).

**Table 1:** Gross characteristics and pancreatic neuroendocrine tumors (PNETs) of Gcgr^−/−^ mice at 10-12 months.

	WT	Heterozygous	Gcgr^−/−^
Numbers observed, n (F/M)	10 (5/5)	25 (14/11)	14 (6/8)
Gross appearance	Fat	Fat	Thin
Body weight (g)	41.9±2.6	39.8±1.4	23.8±0.5*
Visceral fat	Much	Much	Little
Liver weight (mg)	1631±76	1601±46	1335±44*
Pancreas weight (mg)	244±9	256±7	674±30*
Random glucose	157±8	156±6	96±9*
Random glucagon (pg/ml)	109±28	200±16	21165±3391*
Random insulin (ng/ml)	6.7±0.8	5.8±0.4	4.2±0.5
Animals with gross PNETs (≥1 mm)	0	0	12
Animals with microscopic PNETs	0	0	14**
Total number of gross PNETs (≥1 mm)	0	0	36
Size of gross PNETs, median (range), mm	NA	NA	1.4 (1–6.5)
Location of gross PNETs (tail/head)	NA	NA	20/14 (2 unknown)
With lymph node metastasis	NA	NA	0
With liver metastasis	NA	NA	1
Dominant Hormone in gross PNETs	NA	NA	22 tumors from 7 mice were stained
Glucagon			18
Insulin			0
PP			0
SST			0
Ghrelin			0
Mixed			1***
Nonfunctioning			3

All 10 WT and 25 heterozygous mice were euthanized at 12 months. Three Gcgr^−/−^ mice were examined at 10 months, 3 at 11 months, and 8 at 12 months. *p<0.001 comparing Gcgr^−/−^ mice and WT and heterozygous mice as a group. **Both 2 mice with only microscopic PNETs were 10-month-old. ***Co-expressing glucagon and insulin. NA, not applicable. SEM of the mean was given.

The continuous weight gain in WT and heterozygous mice appeared to be due to acquisition of subcutaneous and abdominal visceral fat (Fig 1B). In contrast, the Gcgr^−/−^ mice had little, if any, subcutaneous or abdominal visceral fat. The Gcgr^−/−^ liver was red in color at 2–12 months and weighed slightly lighter (17%) than the WT or heterozygous controls at 12 months (Table 1) which usually appeared fatty. The pancreas was ∼2.5 fold larger in Gcgr^−/−^ mice than in control animals (Fig 1C, Table 1). The patient with the inactivating GCGR P86S mutation was also thin and her pancreas was much larger than that in a control patient, as shown by magnetic resonance imaging (MRI) (Fig 1D).

### Pancreata in younger Gcgr^−/−^ mice

**Figure 2:**
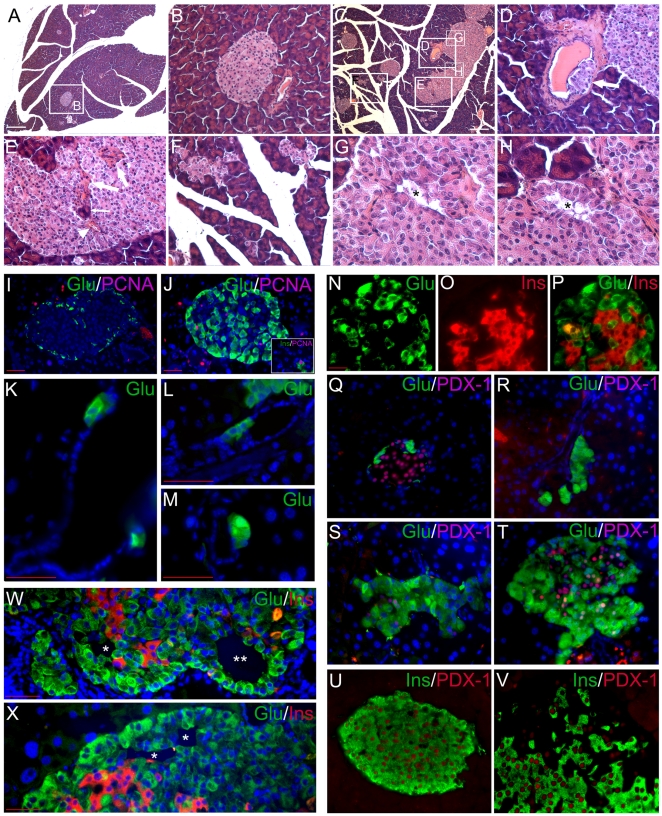
Hyperplastic and dysplastic α cells in Gcgr^**−/−**^ mice at 5-7 months. (A-H) Hematoxylin and eosin (H&E) staining of pancreas of WT and Gcgr^−/−^ mice. (A and B) WT pancreas at low-power (4x) and an islet from A shown in 20x (B). (C) Gcgr^−/−^ pancreas at low-power (4x). (D–H) Five areas of C shown in 20x (D–F) and 40x (G and H). (D) A neogenetic islet (arrow) arising from an exocrine duct; (E) part of a large islet with trapped exocrine ducts (thick arrows), acinar cells (thin arrow), and blood island (arrowhead); (F) singlet endocrine cells and irregularly-shaped islets; and (G and H) intra-islet clearances. The cells around the clearance assume a trabecular growth pattern in H. (I–X) Immunofluorescent staining of pancreas of WT and Gcgr^−/−^ mice. (I and J) PCNA (purple) labeling of WT (I) and Gcgr^−/−^ (J) islet cells. Cells were costained with antibodies for glucagon (green). Inset, Gcgr^−/−^ islet cells costained with antibodies for insulin (green) and PCNA (purple). (K–M) Neogenetic α cells (green) from large (K) and small (L and M) exocrine ducts with formation of a small islet (M). (N–P) Co-production of glucagon (green) and insulin (red) in some Gcgr^−/−^ islet cells. Orange or yellow indicates overlap. (Q–T) WT (Q) or Gcgr^−/−^ (R–T) pancreata were costained for glucagon (green) and PDX-1 (purple or pink). PDX-1 was not present in neogenetic α cells (R) but in some α cells in larger islets (S and T). (U and V) WT (U) or Gcgr^−/−^ (V) pancreata were costained for insulin (green) and PDX-1 (purple or pink). PDX-1 was only present in β cells in WT pancreas but also present in cells without insulin in Gcgr^−/−^ islets. (W and X) Hormonal expression profile of cells around the intra-islet clearances of Gcgr^−/−^ pancreata. The cells without a trabecular growth pattern mostly only express glucagon (*). The cells with a trabecular growth pattern express only glucagon or co-express glucagon and insulin (**). Blue, counterstained with Hoechst 33342 to highlight nuclei. White scale bar, 250 µm; red scale bar, 40 µm. The scale bar in N applies to N–V.

At 2–3 months, the pancreata in Gcgr^−/−^ mice were already larger (306±13 mg) than those in WT (200±14) or heterozygous (185±26) mice (n = 4 in each group) (p<0.01) but otherwise grossly normal. Microscopically, the Gcgr^−/−^ islets were composed of mostly α cells as previously described [15], [16] without dysplasia or PNETs. At 5–7 months, the islets in Gcgr^−/−^ mice (n = 6) exhibited dramatic morphological features which were absent in WT (n = 5) or heterozygous mice (n = 3). The relative islet cell mass was almost 10-fold larger in Gcgr^−/−^ pancreata than that in WT (6.5±0.9% v. 0.7±0.1%, n = 3 in each group, *p*<0.01) (Fig 2A and 2C). Unlike the regularly-shaped islets in WT or heterozygous mice, the Gcgr^−/−^ islets were much larger and irregular in shape (Fig 2C). Some exocrine ducts harbored islet cells in their walls (Fig 2D) and some others were enclosed in islets in a formation classically termed "nesidioblastosis" along with exocrine acinar cells (Fig 2E). Much more exocrine ducts were contiguous with budding islets in Gcgr^−/−^ pancreas than in WT (23.3±1.7% v. 4.2±0.8%, n = 3 in each group, *p*<0.001). Small, irregularly-shaped islet cell clusters were numerous in Gcgr^−/−^ pancreata but virtually absent in WT or heterozygous ones (Fig 2F). Inside larger Gcgr^−/−^ islets, empty spaces lined with islet cells were frequent which sometimes assumed a focal trabecular pattern (Fig 2G, 2F), and most of the lining cells expressed glucagon (Fig 2W and 2X). Some of the empty spaces were filled with red blood cells (Fig 2E). Stand-alone dysplastic islets were rare and usually small. Large (>250 µm) dysplastic islets were not seen. The Gcgr^−/−^ islets were thus hyperplastic and mildly dysplastic at 5–7 months. The Gcgr^−/−^ acinar cells were larger than WT (Fig 2B, 2F). No exocrine tumors or precursor lesions were seen in any genotypes.

### Mechanisms for increased endocrine cell mass in Gcgr^−/−^ mice

As the relative endocrine cell mass was nearly 10-fold larger in Gcgr^−/−^ than in WT pancreata, mechanisms for controlling pancreatic endocrine cell mass such as proliferation, neogenesis, apoptosis, and cell size were examined in 6-7-month-old mice. Cell proliferation was assessed by PCNA or Ki-67 labeling (Fig 2I, 2J). The labeling index was very low and not significantly different in α cells in WT and Gcgr^−/−^ mice (<0.1%) (n = 3 in each group). Surprisingly, in non-α cells (most of which were β cells), the labeling index was significantly higher in Gcgr^−/−^ (34/1156, 2.9%) than in WT (12/1557, 0.7%) mice (*p*<0.001) (Fig 2J and inset). α cell neogenesis was assessed by 2 methods. Singlet and doublet α cells were frequent in Gcgr^−/−^ but absent in WT animals (49±7 v. 0±0/cm^2^, *p*<0.001) (Fig 2B, 2F). Exocrine ducts harboring glucagon-positive cells were also more frequent in Gcgr^−/−^ (12/478, 2.5%) than in WT (1/294, 0.3%) pancreata (*p*<0.05) (Fig 2K-2M). The apoptotic rates were very low in both Gcgr^−/−^ and WT endocrine cells and were not significantly different (<0.1% in both groups). The α cells were significantly larger in Gcgr^−/−^ than in WT pancreata (133±5 v. 54±2 µm^2^, *p*<0.001), as well as their nuclei (45±1 µm^2^ v. 33±1 µm^2^, *p*<0.001) (for direct comparison, see Fig 2I and 2J).

### Abnormal α cell differentiation in Gcgr^−/−^ mice

Unlike the WT or heterozygous controls, some Gcgr^−/−^ islet cells produced both glucagon and insulin (Fig 2N-2P), as previously shown in another genetic background of mice with deficient Gcgr [16]. As pancreatic and duodenal homeobox 1 (PDX-1) directs lineage specification to insulin-producing in adult animals and is expressed in glucagon-positive cells of young Gcgr^−/−^ animals [17], we studied PDX-1 expression in Gcgr^−/−^ islets at 5–7 months. In WT islets, nuclear PDX-1 and cytoplasmic insulin were strictly co-expressed in all 596 cells examined and no glucagon-positive cells expressed PDX-1 (Fig 2Q, 2U). In Gcgr^−/−^ islets, PDX-1 was not seen in any neogenetic α cells (those budding from exocrine ducts), but some α cells in larger islets clearly expressed PDX-1 (Fig 2R-2T). Of 656 Gcgr^−/−^ cells expressing insulin, all (100%) simultaneously expressed PDX-1, indicating that PDX-1 is necessary for insulin expression in Gcgr^−/−^ islets. Of 760 Gcgr^−/−^ islet cells expressing PDX-1, 656 (86.3%) expressed insulin but 104 (13.7%) did not, indicating that PDX-1 expression is not sufficient to drive insulin expression in Gcgr^−/−^ mice. There was no significant co-expression of glucagon and pancreatic polypeptide or glucagon and somatostatin. We did not find significant increase in the number of somatostatin-expressing cells as previously reported in younger animals [16], [17] likely due to differences in age or genetic background.

### PNETs in Gcgr^−/−^ mice

**Figure 3:**
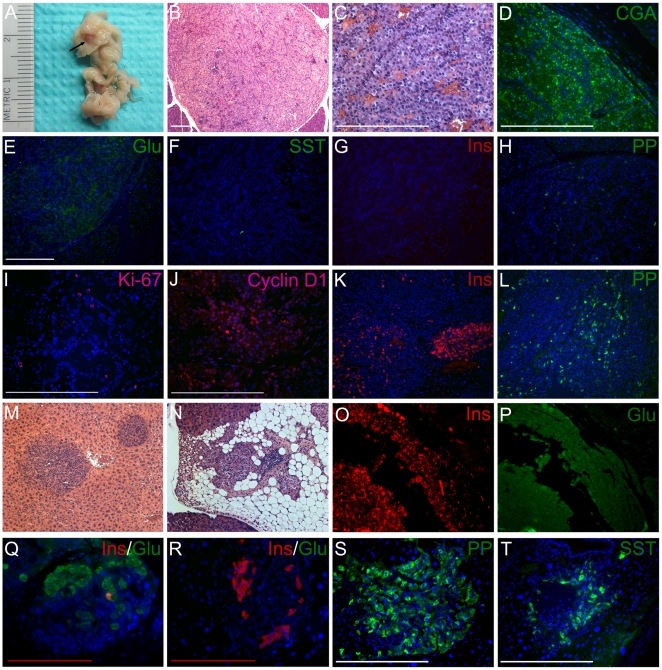
Pancreatic neuroendocrine tumors (PNETs) and dysplastic endocrine cells in Gcgr^**−/−**^ mice at 10-12 months. (A–P) PNETs in Gcgr^−/−^ mice. (A) A PNET (arrow) inside the tail of a fixed whole pancreas from a 12-month Gcgr^−/−^ mouse. (B and C) H&E staining of the tumor in A (4x and 20x, respectively). (D) Immunostaining for chromogranin A (CGA, green) of the tumor in A. (E–L) PNETs from 10-12-month Gcgr^−/−^ mice were immunostained for glucagon (Glu) (E, green), somatostatin (SST) (F, green), insulin (Ins) (G and K, red), pancreatic polypeptide (PP) (H and L, green), Ki-67 (I, pink), and cyclin D1 (J, pink). (M) H&E staining of liver with metastatic PNET foci. (N) Focal fat replacement of exocrine pancreas with preservation of dysplastic islets. (O and P) A PNET co-expressing insulin (O, red) and glucagon (P, green). (Q-T) Dysplastic endocrine cells from 12-month Gcgr^−/−^ mice immunostained for islet hormones. (Q and R) Pancreas from Gcgr^−/−^ mice was doubled stained for insulin (red) and glucagon (green). Orange or yellow denotes overlap of red and green fluorescence. (S and T) Pancreas from Gcgr^−/−^ mice was stained for pancreatic polypeptide (S) or somataostatin (T) (both green). Blue represents nuclei in fluorescent images. White scale bar, 250 µm; red scale bar, 125 µm. The scale bar in E applies to E–P except for I and J.

At 10–12 months, gross PNETs (≥1 mm) were clearly detected in most (12/14) Gcgr^−/−^ pancreata but not in any WT (0/10) or heterozygous (0/25) ones (Table 1 and Fig 3). Within each genotype, the pancreas morphology and other genotype-specific phenotypes were similar between both sexes so that male and female mice were analyzed together. These tumors were denser than normal pancreas and highly vascularized (Fig 3A). They were found at all parts of the pancreas but slightly more frequently at the tail half. Larger tumors (≥3 mm) tended to adhere to the surrounding organ such as the small intestine, colon, or spleen. Enlarged peripancreatic lymph nodes were commonly seen, regardless of the Gcgr genotype. Gross liver metastasis was uncommon and only present in a miliary form in one mouse with a very large pancreatic tail PNET (6.5-mm). Histologically, the PNETs were composed of uniform cells arranged in a trabecular pattern, consistent with neuroendocrine tumors (Fig 3B, 3C). Blood islands and fibrostromal tissue were evident in these tumors but necrosis was rare. Larger tumors usually had a capsule which was often invaded by the tumor cells. All the tumors were positive for neuroendocrine marker chromogranin A (Fig 3D). Most PNETs expressed glucagon except for a few which did not express any islet hormones (Table 1 and Fig 3E). Insulin, pancreatic polypeptide, and somatostatin were sporadically present in most PNETs (Fig 3F-3H). In some tumors, insulin or pancreatic polypeptide were focally present but the number of cells in the focus was small (Fig 3K, 3L). A mixed glucagonoma/insulinoma was present in one mouse although it had insulin levels comparable to those of other Gcgr^−/−^ mice (Fig 3O, 3P). Mitotic figures were rare and the average Ki-67 labeling index was 1.7% (range 1.0-3.6%) (Fig 3I), similar to that of the nonfunctioning PNET in the patient with inactivating GCGR mutation [18]. Cyclin D1 was only focally positive in a large PNET (Fig 3J). Histological examinations of liver sections of the mouse with suspected miliary liver metastases identified neuroendocrine tumor foci (Fig 3M). Rarely, fatty replacement of pancreas parenchyma was seen with preservation of dysplastic islets in Gcgr^−/−^ pancreata but not in WT or heterozygous ones. Two 10-month Gcgr^−/−^ mice only harbored micro-PNETs (<1 mm). Thus gross or micro-PNETs were present in all Gcgr^−/−^ mice at 10–12 months. Careful examination of multiple pancreatic sections of 10 WT and 25 heterozygous 12-month mice did not reveal any dysplastic islets or PNETs (micro- or gross). We did not find any exocrine tumors, carcinoma-in-situ, or gross carcinomas in any animals of the 3 genotypes at 12 months.

**Figure 4:**
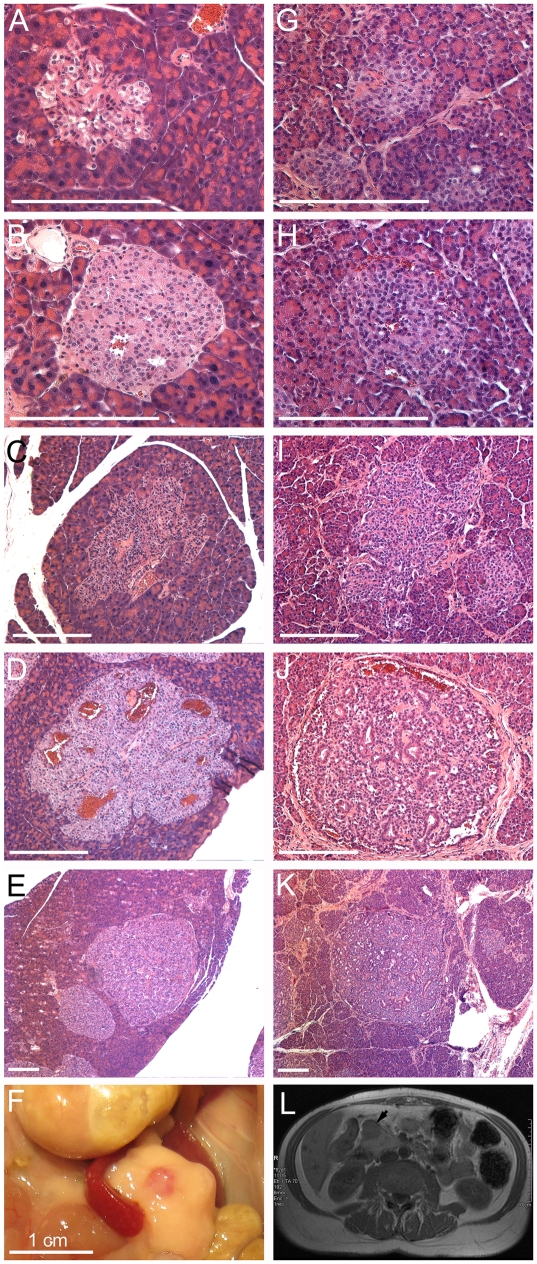
The Gcgr^**−/−**^ mice recapitulate all stages of PNETs in a human patient with a homozygous inactivating mutation of glucagon receptor. (A–E, G–K) H&E-stained pancreatic sections. (F) A posterior PNET revealed after the pancreas was flipped after euthanasia. (L) Abdominal MRI of the patient. (A–F) From 12-month-old Gcgr^−/−^ mice. (G–L) From the 60-year-old patient. Dysplastic islets with stromal component (A, G) or with blood islands (B, H), microadenomas about 300 µm (C, I), 600 µm (D, J), or 1 mm (E, K), and gross tumors (F, L) are all present. Scale bar, 250 µm until otherwise indicated. Panel L is from reference 18.

Dysplastic islets were numerous in the non-tumor part of pancreas of the 12-month Gcgr^−/−^ mice but were not found in any 12-month WT or heterozygous pancreas. In contrast to the dysplastic islets at 5–7 months, the dysplastic islets at 12 months were more often stand-alone and larger. In rare instances, it was practically impossible to tell a large dysplatic islet from a micro-PNET. Although most of the dysplastic islets predominantly expressed glucagon and insulin, some islet cells did not express either glucagon or insulin and others expressed focally pancreatic polypeptide or somatostatin (Fig 3Q-3T), suggesting chaotic differentiation of the dysplastic islet cells. Similar dysplastic islets were evident in the non-tumor pancreas of the patient which also harbored microscopic PNETs of various sizes (Fig 4). Thus all stages of PNET tumorigenesis including hyperplasia, dysplasia, microscopic PNET, and gross PNET were similarly observed in the 10-12-month-old Gcgr^−/−^ mouse pancreata and the pancreas of the patient with inactivating GCGR mutation (Fig 4).

### Molecular mechanisms for PNET pathogenesis in GCGR^−/−^ mice

**Figure 5:**
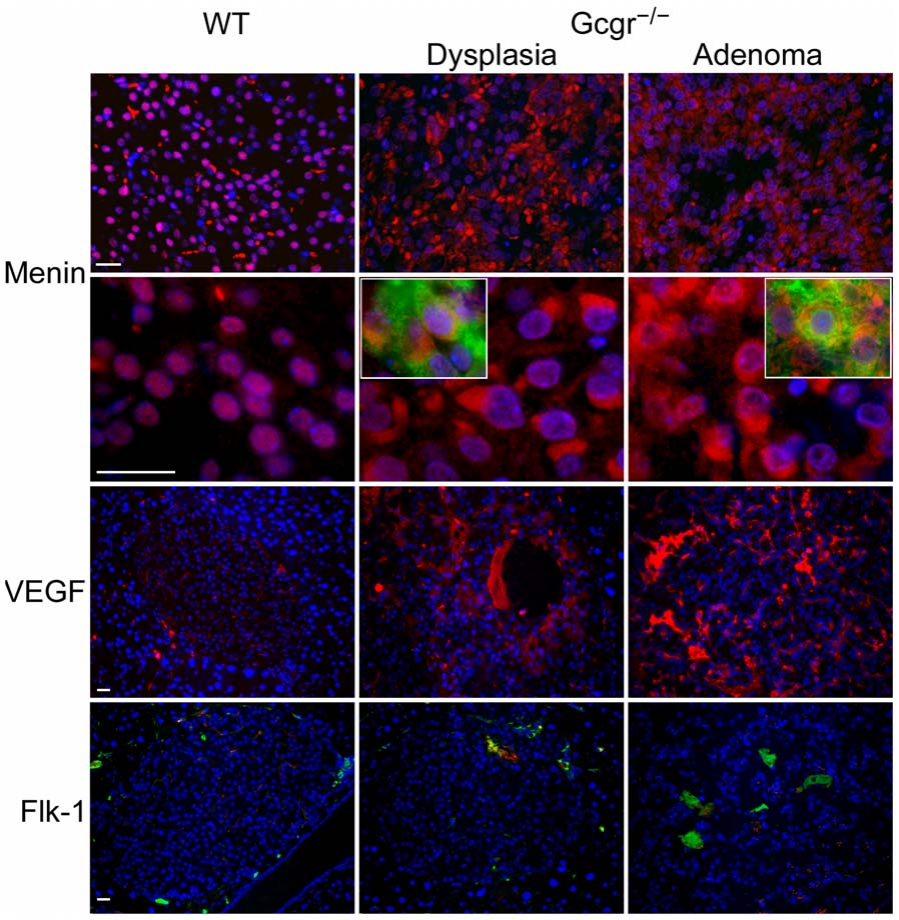
Abnormal menin expression and angiogenesis in Gcgr^**−/−**^ endocrine pancreas. Pancreatic sections from 12-month-old WT or Gcgr^−/−^ mice were immunostained for menin (red), vascular endothelial growth factor (VEGF, red), and VEGF receptor Flk-1 (green). All slides were counterstained with Hoechst 33342 for highlighting nuclei (blue). Insets, dysplatic islets and adenomas in Gcgr^−/−^ pancreas were costained for menin (red) and glucagon (green). Yellow or orange indicates colocalization of menin and glucagon. Bar, 20 µm.

The molecular pathogenesis of PNETs is poorly understood [21], [22]. We stained WT and Gcgr^−/−^ pancreata for p53, mdm2, and pRB and did not find significant differences in their expression and localization (data not shown). Since menin is commonly mutated or abnormally localized or expressed in sporadic PNETs [23], [24], we immunostained menin in WT and Gcgr^−/−^ pancreata and in Gcgr^−/−^ PNETs. In WT islets, menin was expressed and localized to the nuclei (Fig 5) at 12 months. In Gcgr^−/−^ pancreas at similar age, menin was expressed at about the same levels as in WT islets assessed with immunostaining but its localization was mainly cytoplasmic in both dysplastic islets and in PNETs. Similar aberrant subcellular localization was seen in Gcgr^−/−^ islets at as early as 5 months, even in apparently normal-appearing islets (data not shown). As menin inhibits angiogenesis at least partially through suppressing vascular endothelial growth factor (VEGF) [25], we studied VEGF expression by immunostaining (Fig 5). VEGF expression was very low in WT islets but was clearly upregulated in Gcgr^−/−^ dysplastic islets and even more so in PNETs. The expression of Flk-1, a major VEGF receptor, was minimal in WT islets but was seen in the empty spaces filled with red blood cells and enclosed by dysplastic islet cells overexpressing VEGF. Flk-1 expression levels were highest in PNETs where Flk-1 was localized to the spaces enclosed by tumor cells growing in a trabecular manner.

## Discussion

As glucagon signaling plays important roles in type 2 diabetes pathogenesis, it is reasonable to experiment with inhibiting glucagon signaling in an attempt to improve diabetes control [2], [6], [7]. Although glucagon signaling inhibition clearly shows various metabolic benefits for diabetes treatment in animal (mostly murine) models, hyperglucagonemia and α cell hyperplasia cast doubt on the safety of that approach [8]-[14]. Our recent description of a novel human disease (Mahvash disease) with hyperglucagonemia, α cell hyperplasia, and pancreatic neuroendocrine tumors (PNETs) associated with a homozygous inactivating GCGR mutation suggests that the pancreata of mice and humans may respond similarly to glucagon signaling inhibition [18], [19]. In this study, we have therefore studied the phenotypes of Gcgr-deficient (Gcgr^−/−^) mice to test the hypothesis that defective glucagon signaling causes PNETs. Our results demonstrate that PNETs develop in Gcgr^−/−^ mice by 10–12 months with 100% penetrance, confirming the hypothesis.

Similar to that in other murine PNET models, especially the more physiological ones with various forms of heterozygous Men1 deletion [26]–[33], the pathogenesis of PNETs in Gcgr^−/−^ mice follows a morphologically distinct multistage pathway. In younger animals (2–3 months), islet cell hyperplasia predominates without clear dysplasia ([16] and this current study). In middle-aged animals (5–7 months), islet dysplasia becomes evident. In older animals (10–12 months), islet dysplasia is pervasive and microscopic or gross PNETs emerge from the dysplastic background. The low incidence of liver metastasis in Gcgr^−/−^ mice probably reflects the low-grade nature of their PNETs. In the Gcgr^−/−^ mice, the islet cells are also hypertrophic at all stages. The remarkable similarity among the multiple murine PNET models suggests similar mechanisms for PNET pathogenesis in those models.

The hyperplasia in mice with menin deletion is at least partially caused by loss of one menin allele which is known to allow excess cell proliferation [32], [34]. In the Gcgr^−/−^ mice, α cell hyperplasia is intuitively easily comprehensible as loss of negative feedback inhibition of glucagon signaling, but the molecular mechanisms are not clear. The α cells appear to be exquisitely responsive to inhibition of glucagon signaling. Inhibition of Gcgr expression by Gcgr antisense oligonucleotides, abolishing glucagon production by deletion of prohormone convertase 2 gene or preproglucagon gene, small-molecule GCGR antagonists, GCGR-antagonizing antibodies, and liver-specific deletion of Gsα all result in α cell hyperplasia as seen in the Gcgr^−/−^ mice and the patient with Mahvash disease [10]–[16], [18]–[20], [35]–[37]. As α cell hyperplasia can be due to increased proliferation, neogenesis, and decreased apoptosis [38], we have studied each of these mechanisms in this study. We did not find increased α cell proliferation but instead, found increased non-α cell proliferation. As have been shown previously and in this study, some α cells in Gcgr^−/−^ mice express PDX-1 and α cells can potentially be transdifferentiated into β cells [17], [33], [39]. Increased proliferation thus contributes to non-α cell hyperplasia but not α cell hyperplasia. As evidence of significant α cell neogenesis is pervasive in Gcgr^−/−^ pancreas and apoptosis is barely detectable in all genotypes, neogenesis is likely the predominant mechanism for α cell hyperplasia in Gcgr^−/−^ pancreas. How glucagon signaling inhibition induces α cell neogenesis is not clear but nestin, a neural stem cell marker expressed both in exocrine and endocrine cells, is upregulated by decreased glucagon signaling and may mediate α cell neogenesis [40]. Remarkably, α cell hyperplasia associated with inhibition of glucagon signaling is mostly reversible within 2 months after the inhibition is removed [11], [41], [42], suggesting a possible therapeutic window of opportunity for patients with Mahvash disease at the hyperplastic stage. As α cell hyperplasia induced by glucagon signaling inhibition occurs similarly in mice and humans, it is likely mediated by similar mechanisms. The Gcgr^−/−^ mice thus could be a very suitable model for addressing the molecular mechanisms of the negative feedback control on α cells exerted by glucagon signaling.

The transition from islet hyperplasia to dysplasia and PNETs is not well understood [32]. It is not even clear if hyperplasia is required to develop dysplasia and PNETs [43]. The commitment to tumorigenesis probably occurs at the formation of islet dysplasia [32]. Our data cannot directly address whether hyperplasia is required for PNET tumorigenesis but suggest some clues to PNET pathogenesis in Gcgr^−/−^ mice. First, the cells in dysplastic islets are mostly α cells and most PNETs are glucagonomas, indicating that the PNETs are likely derived from α cells. Although transdifferentiation from non-α islet cell types is possible [17], [39], it is unlikely as there is no evidence of preceding hyperplasia, dysplasia, or PNET of another islet cell type. Second, the hyperplastic α cells in Gcgr^−/−^ mice harbor abnormal transdifferential capacity to develop into mixed α/β cell type or lose hormonal expression, suggesting that they may be prone to genomic or epigenomic instability, a harbinger of tumorigenesis. Third, the abnormal subcellular menin localization and abnormal angiogenesis in dysplastic islets and PNETs imply that deranged menin expression plays a role in PNET pathogenesis in Gcgr^−/−^ mice. Menin localization is abnormal in up to 80% of sporadic PNETs and menin mutations are common in such tumors as well [24], [25]. How GCGR deletion leads to abnormal menin localization is not clear but it may be due to spontaneous menin mutations as shown in human sporadic PNETs [25].

Although the Gcgr^−/−^ mice and other murine models are consistent in recapitulating the pathogenesis of their respective human diseases (Mahvash disease and multiple endocrine neoplasia type 1), they might have limited value in understanding the pathogenesis of sporadic human PNETs which do not clearly arise from a background islet hyperplasia and dysplasia [44]–[46]. The Gcgr^−/−^ mice, however, do provide insights into the natural history of Mahvash disease in humans. For example, the indistinguishable pancreas morphology of heterozygous and WT mice provides reassurance that humans with heterozygous GCGR P86S mutation are likely free of the disease, a conclusion that could only be inferred by observing patients with confirmed or presumed heterozygous P86S mutation. The Gcgr^−/−^ mice also indicate that PNETs, the most clinically significant component of Mahvash disease, take a long time to form, which should be considered when deciding on the initial age for starting clinical screening of such tumors. In addition, the similar pancreas phenotype in both sexes of Gcgr^−/−^ mice suggests that Mahvash disease affects men and women equally. Finally, the significant hypoglycemia in older Gcgr^−/−^ mice suggests that older patients with Mahvash disease should be carefully monitored for glucose levels, especially if inflicted with an acute concomitant disease.

The Gcgr^−/−^ mice and the human patient with Mahvash disease strongly suggest that complete inhibition of glucagon signaling as a therapeutic approach to treat type 2 diabetes may have limited clinical potential due to safety concerns. Indeed, the Gcgr^−/−^ mice have metabolically desirable features, indicating that some degree of glucagon receptor antagonism is beneficial to glycemic control [15], [16], [47]. Unfortunately, those metabolic benefits are offset by serious risks in older animals. Apart from the relatively minor risks of lower female fertility and decreased vision [17], [48], PNETs develop with 100% penetrance by 10–12 months as shown in this study. We have thus demonstrated that defective glucagon signaling cause PNETs in mice with Gcgr deletion and in humans with inactivating GCGR mutation (this current study and [19]). It is yet to be determined if other means of glucagon signaling inhibition also causes PNETs. As both mice and humans with defective GCGR exhibit almost identical phenotypes to those induced by glucagon signaling inhibition by other mechanisms [8]–[16], [18], [19], it is reasonable to propose that complete pharmacological inhibition of glucagon signaling will result in similar phenotypes, which could ultimately result in PNETs over long-term treatment. On the other hand, since mice and humans with defective GCGR are under complete glucagon signaling inhibition from birth, it is possible that PNETs may not develop in subjects with intact glucagon receptor who receive pharmacological and incomplete inhibition of glucagon signaling initiated during adulthood as intended.

In summary, we have demonstrated that PNETs develop in older Gcgr^−/−^ mice with 100% penetrance and the Gcgr^−/−^ mice may be used as a model of human PNETs. Our results suggest that complete inhibition of glucagon signaling may not be a safe approach to treat diabetes.

## Materials and Methods

### Animals

Gcgr^−/−^ mice were generated and provided to us by Pfizer Global Research and Development. They were derived from a DBA1/LacJ ES cell line microinjected into C57BL/6 blastocyst stage embryos [15]. Male chimeric offspring were then bred to DBA1/lacJ females. Heterozygous offspring from these matings were intercrossed for two generations to produce the two heterozygous males and two heterozygous females that were used to produce all the mice used in this study. In contrast to the mice used in reference 15 which were backcrossed onto C57BL/6J, the mice used in this study were purebred DBA1/lacJ. All animals used in this study were offspring of heterozygous parents, and WT and heterozygous littermates were used as controls for Gcgr^−/−^ mice. Animals were housed in a 12-h dark/light cycle and fed with standard chow ad libitum. Mice were genotyped by PCR methodology based on ear notch or tail genomic DNA. Glucose was measured by a glucometer. Mice were sacrificed by CO_2_ asphyxiation, and pancreata inspected, harvested, weighed, and fixed in 10% neutral formalin and embedded in paraffin. Experiments were reviewed and approved by the Cedars-Sinai Institutional Animal Care and Use Committee (project # IACUC002552).

### Hormonal assays

Blood was obtained through cardiac puncture after euthanasia. Serum or plasma was prepared and glucagon was measured by radioimmunoassay (Millipore, Billerica, MA) and insulin by ELISA (Crystal Chem, Downers Grove, IL). Measurements were carried out according to manufacturers' recommendations.

### Patient

The patient with a homozygous inactivating P86S mutation of glucagon receptor has been described previously [18], [19]. The patient gave informed written consent for the study which was approved by the Cedars-Sinai Institutional Review Board (project # Pro00016895).

### Histology

The mouse pancreas was carefully placed in the tissue block to make sure that the head, body, and tail were parallel to the cut surface of the block. Some pancreas tissue blocks derived from 6-7-month-old WT and Gcgr^−/−^ mice were from Pfizer. Tissue slices every 100 microns were saved. Ten 5-µm sections per pancreas were stained with hematoxylin and eosin (H&E) and histology was reviewed by two of the authors (one physician/scientist with extensive experience in pancreas histology and one pathologist specializing in gastrointestinal pathology) who agreed with the findings. Dysplastic islets were clearly different from normal islets in that the former had blood islands and significant stroma. Trabecular cell growth pattern is the most distinct and defining feature of pancreatic neuroendocrine tumors (PNETs). Larger PNETs had capsules as well. *Morphometry of endocrine cell mass* was achieved by scanning hematoxylin and eosin-stained slides as previously described [49]. Endocrine cell mass was calculated as the average of the total area of endocrine cells divided by the total area of the pancreatic section. The patient's surgical pancreas sample from a partial pancreatectomy was studied as before [18], [19].

### Immunofluorescent staining

Paraffin pancreatic 5-µm sections were deparaffinized, rehydrated, and incubated with the appropriate primary and secondary antibodies as previously described [49]. The rabbit anti-glucagon, anti-pancreatic polypeptide, and anti-somatostain, and guinea pig anti-insulin antibodies (Dako, Carpinteria, CA), and mouse anti-Flk-1 (Santa Cruz Biotechnology, Santa Cruz, CA) were used directly without antigen retrieval. The sections were heated in an antigen retrieval buffer (Dako) for 40 minutes before incubation with the following primary antibodies: mouse anti-PCNA, goat anti-Ki-67, mouse anti-p53, mouse anti-mdm2, rat anti-pRB, and goat anti-menin (Santa Cruz), rabbit anti-cyclin D1, rabbit anti-chromogranin A, and guinea pig anti-PDX-1 (Abcam, Cambridge, MA), rabbit anti-menin (Bethyl Laboratories, Montgomery, TX), and mouse anti-VEGF (Novus Biologicals, Littleton, CO). Rhodamine- or FITC-labeled secondary antibodies were used. For double staining, primary antibodies from different species were used, followed by appropriate combination of secondary antibodies. All sections were also stained with Hoechst 33342 to highlight the cell nuclei and chromosomes.

### Apoptosis assay

Pancreatic sections were heated in an antigen retrieval buffer followed by incubation with FITC-terminal deoxynucleotidyltransferase-mediated deoxyuridinetriphosphate nick end labeling (TUNEL) reagent (Roche, Indianapolis, IN) at 37 C for 1 hour [49]. The sections were then stained with rabbit anti-glucagon and rhodamine-labeled anti-rabbit antiserum, and counter-stained with Hoechst 33342. Apoptosis was confirmed if a cell's nucleus is both TUNEL-positive and fragmented and condensed as revealed by Hoechst 33342.

### Microscopy

Stained pancreatic sections were examined using a Nikon Eclipse TE200 or an Olympus IX2-SP fluorescence microscope equipped with the appropriate optic filters. Pictures were captured using a SPOT (Diagnostic Instruments, Sterling Heights, MI) or a MegnaFire (Olympus, Center Valley, PA) digital camera and processed with Adobe Photoshop (San Jose, CA).

### Data analysis

For most studies, at least 5 animals of similar age were included in each group. For quantitative analysis, at least 3 animals were used in each group. For cell and nuclear size measurements, a total of 100 random cells from 5 animals were used. Student t test was used to compare the means of continuous parameters. Fisher's exact test was used to compare frequencies.
